# Early Effects of Information Revolution Interventions on Health Information System Performance in Ethiopia

**DOI:** 10.9745/GHSP-D-23-00513

**Published:** 2024-12-20

**Authors:** Barbara Knittel, Heather M. Marlow, Afrah Mohammedsanni, Abebaw Gebeyehu, Hiwot Belay, Wubshet Denboba

**Affiliations:** a JSI Research &Training Institute, Inc., Washington, DC, USA.; b JSI Research & Training Institute, Inc., Addis Ababa, Ethiopia.

## Abstract

Health information systems (HISs) are essential to a country’s health system as they provide critical support to health policymaking, management, financing, and service delivery. A well-functioning HIS should produce timely and reliable data that are available and easily accessible to decision-makers throughout the health system. Ethiopia has transitioned from a fragmented, paper-based health management information system (HMIS) to a harmonized, digital system used at points of collection and service delivery.

In 2016, the Federal Ministry of Health (MOH) launched the Information Revolution (IR), a transformative agenda aimed at enhancing the culture of data use, scaling priority HIS tools and systems, and strengthening HIS governance. Between 2016 and 2022, the MOH, Data Use Partnership, and other partners implemented a series of IR interventions that supported this agenda. These interventions included deploying and harmonizing digital HIS systems; strengthening HIS leadership, coordination, and governance; implementing the IR pathway strategy; enhancing capacity through supportive supervision, mentorship, and training; and improving performance monitoring teams. This article aims to synthesize the key HIS interventions implemented in Ethiopia as part of the IR and document the effects of these interventions on HIS performance.

Early studies indicate promising improvements in HIS performance across health facilities in Ethiopia. However, challenges remain. To ensure sustainable progress, it is essential to continue addressing key challenges, such as system interoperability, HIS workforce, and capacity for data use at all levels. By building on the successes of the first Health Sector Transformation Plan and addressing these gaps, Ethiopia can advance its vision of a robust, data-driven health system capable of improving health outcomes and driving evidence-based decision-making.

## Abstract (precis)

Ethiopia’s shift to a digital health information system under the Information Revolution has laid a strong foundation for improved data quality. To fully realize the potential of this transformation, ongoing commitment to addressing key challenges in system integration and capacity-building is vital.

## Teaser

**Key Findings:**

- Ethiopia’s shift to a unified digital health management information system (HMIS) and the launch of the Information Revolution have led to significant improvements in data quality and use.
- Key interventions included harmonizing digital systems, enhancing governance and leadership, capacitating the health information system (HIS) workforce to use data, and strengthening performance monitoring teams.
- Notable progress includes increased data completeness and accuracy at health facilities and better use of data for decision-making. However, challenges such as system interoperability and workforce shortages remain.
- Early studies show promising improvements, but sustained progress will require continued efforts to address gaps in system integration and expand workforce capacity.

**Key Implications:**

- Continued investment in digital HIS infrastructure and policy alignment is essential to ensure seamless data integration and enhance system functionality.
- Strengthening the HIS workforce through targeted training and mentorship is crucial for maintaining high standards of data quality and effective use.
- Persistent challenges, such as system interoperability and workforce shortages, need to be addressed to ensure that improvements are sustained and that health data continues to drive evidence-based decision-making and improved health outcomes.

## BACKGROUND

Health information systems (HISs) are essential to a country’s health system as they provide critical support to health policymaking, management, financing, and service delivery. When effective, HISs contribute directly and indirectly to improved quality, efficiency, and effectiveness of health services and accelerate desired health outcomes.^1^ A well-functioning HIS provides timely, reliable data that are available and easily accessed by decision-makers throughout the health system. While data quality is one aspect of a functioning HIS, the ability and interest of health workers to analyze and use routine data for decision-making is equally important.^2^ The Performance of Routine Information System Management (PRISM) framework^3^ identifies key behavioral, technical, and organizational factors influencing data quality and use. These include perceived personal benefits in using data, organizational culture toward data, availability of a skilled workforce to process and analyze data, availability of resources, and adequate leadership that promotes quality.^4^

Ethiopia has transformed its health management information system (HMIS) from a fragmented, paper-based system to a harmonized digital platform. From 2006 to 2016, the Federal Ministry of Health (MOH) reformed and expanded its national HMIS to health facilities across the country.^5^ During this time, the MOH revised HMIS indicators and reporting mechanisms to ensure alignment across HISs.^5^^,^^6^ In 2010, the MOH introduced the Community Health Information System (CHIS) to monitor the quality of health service provision at the community level.^7^ In 2016, the MOH launched the Information Revolution (IR) as part of the Health Sector Transformation Plan (HSTP-I).^8^ The focus of this agenda, outlined in the national IR Roadmap (2016–2020), identified 3 pillars: (1) enhancing the culture of information use for decision-making, (2) implementing and scaling up a prioritized HIS and tools, and (3) strengthening HIS governance.^9^^,^^10^

**FIGURE 1:**
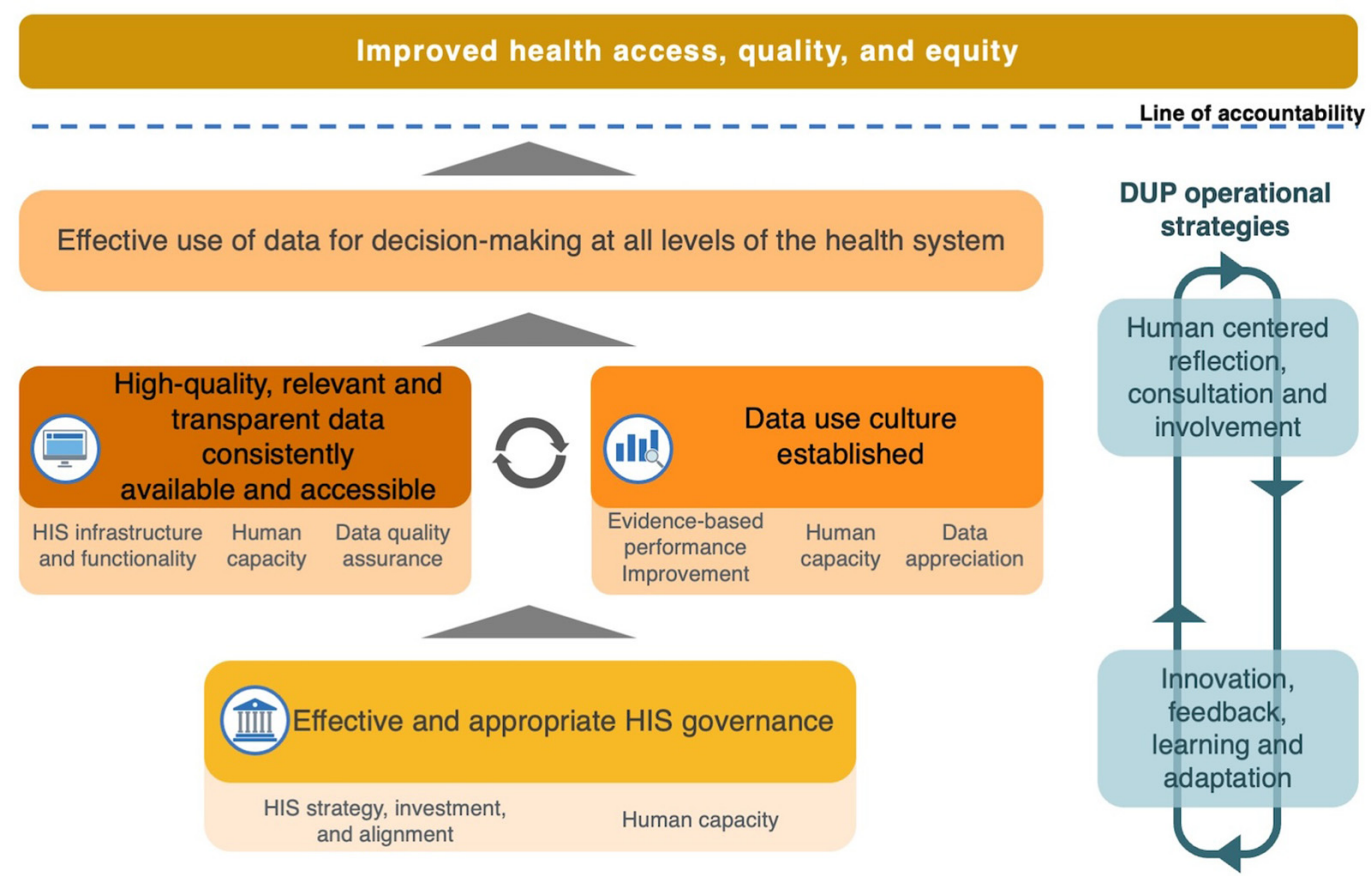
Information Revolution Theory of Change in Ethiopia Abbreviations: DUP, Data Use Partnership; HIS, health information system.

To support the implementation of the IR, the Data Use Partnership (DUP), a project co-created by the MOH in collaboration with JSI Research & Training Institute, Inc. (JSI) and funded by the Bill & Melinda Gates Foundation, was implemented to support and strengthen HIS efforts and operationalize its vision. Between 2016 and 2022, the MOH, DUP, and other partners implemented interventions in HIS digitization, governance and leadership, and data quality and use (Figure 1). This article aims to synthesize the key HIS interventions implemented in Ethiopia from 2016 to 2022 as part of the IR and reflect on the outcomes of key program interventions and their effects on HIS performance.

## BASELINE HEALTH INFORMATION SYSTEM PERFORMANCE

PRISM baseline assessments were conducted between 2018 and 2020, before the start of many IR interventions, to establish estimates of key HIS performance indicators at the national and regional levels and inform targeted interventions needed in different contexts. Indicators included data quality (completeness, timeliness, accuracy, and consistency); data use (production, management practices, and dissemination); and technical, organizational, and behavioral factors that affect performance (resource availability, staff competency and motivation, and HIS infrastructure).^3^

Regional assessments in Addis Ababa, Harari, Somali, and Benishangul Gumuze indicated that dimensions of data quality were low across the health system.^11^^–^^14^ For example, Haftu et al. found that the quality of routine data collected at 33 health centers in Addis Ababa fell outside of the acceptable tolerance ranges, with only 42% of facilities submitting data on time and just 16% of health centers’ reported data falling within the acceptable threshold of accuracy (90%–110%).^11^ Similarly, a data quality study conducted in Harari region indicated that 49% of assessed facilities had data quality outside of acceptable tolerance ranges (accuracy: 90%–110%, completeness: ≥85%, timeliness: ≥85%).^12^

Several regional assessments reported that data quality and routine use of data was low.

The use of routine data was also low across many published regional assessments. A study among 408 health workers in Addis Ababa found that only 37% were using routine health information for decision-making, and just over 50% had acceptable skills in data collection, analysis, information presentation, and use.^15^ In 2 districts of Northwestern Ethiopia, only 39% and 46% of health facilities demonstrated above average data use across 5 variables: reception of feedback, evidence-based decision-making, indicator identification, coverage calculation, and target versus achievement calculation.^16^

Many of these studies also identified factors that negatively affected data quality and use, such as inadequate supportive supervision and feedback mechanisms, insufficient HIS infrastructure, low leadership engagement, and a lack of commitment to data use.^11^^–^^15^ Additionally, individual factors, including poor motivation, limited data skills, and low staff commitment, were found to further hinder data quality and use.^10^^,^^16^^–^^18^

## KEY HEALTH INFORMATION SYSTEM INTERVENTIONS

### Foster a Culture of Data Use Through the Information Revolution Pathway

**FIGURE 2:**
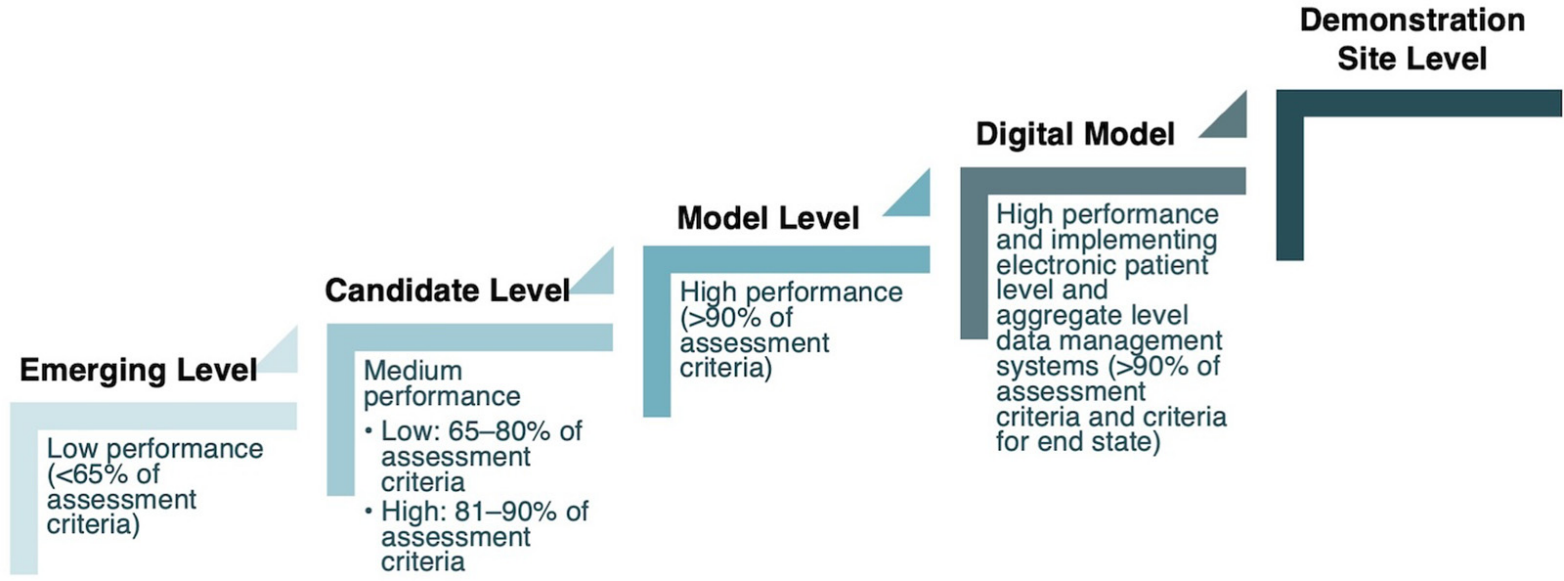
Information Revolution Pathway Scoring Criteria Used to Evaluate Facilities and Woredas in Health Information System Performance

The first IR pillar fosters a nationwide culture of data use as the main link between health data and improved health services. To promote data quality and use across all levels, the MOH introduced the Information Revolution Pathway (formerly the Connected Woreda Strategy), a tiered framework for facilities and woredas to reach high standards in data quality, use, and digital connectivity.^19^ A key part of this strategy was to evaluate and score facilities and woredas against a common set of criteria related to HIS infrastructure, data quality, and administrative and clinical data use.^19^ Facilities and woreda that met the highest standards—capable of accessing, using, and sharing data offline—earned the designation of “model level.” These model units then progress to “digital model” status by enabling online data access and transmission (Figure 2). After categorization, facilities received targeted HIS interventions, such as capacity-building and mentorship, to improve their performance. Their progress was periodically reassessed and updated based on improvements in their HIS performance scores.^19^

### Improve Data Quality and Use Through Capacity-Strengthening and Mentoring

Another IR strategy to improve data quality and use was to invest in capacity-strengthening by building the skills and knowledge of managers and decision-makers to oversee data collection, management, and interpretation for action. One way this was implemented was through the Capacity Building Mentorship Program (CBMP). The MOH launched this program in partnership with 6 local universities: Addis Ababa University, Haramaya University, Hawassa University, Jimma University, Mekelle University, and University of Gondar. The program aimed to improve HIS performance by fostering collaboration between academic institutions and local health administrations by building ownership and capacity in both sectors.

Through the CBMP, 6 universities in Ethiopia conducted HIS in-service training course to build the capacity of the HIS workforce in data quality improvement and data use.

As part of the CBMP, universities introduced HIS in-service training courses for health workers and developed degree programs to prepare graduates for the HIS workforce. By 2022, 814 students had enrolled in these programs. The program also supported 6 doctoral and 43 master’s students in conducting operations research at participating CBMP health facilities.^20^ Additionally, universities provided technical assistance to regional health bureaus in 36 woredas, including supportive supervision, mentorship, on-site coaching and training in data quality improvement, and cross-site experience sharing. Other capacity-building efforts included integrating HIS courses into pre-service trainings, providing in-service training on HIS topics for the MOH, regional health bureaus, zonal health departments, and health facility staff, as well as conducting supportive supervision and mentorship to health institutions. Furthermore, 74 CBMP facilities were designated as “learning and demonstration sites,” receiving bimonthly structured mentorship and on-site, needs-based training to enhance improving HIS capacity and competency.^8^

### Enhance Performance Monitoring Teams to Effectively Use Data

Another key intervention under pillar 1 was to enhance the functionality of performance monitoring teams (PMTs) at both the facility and woreda levels. These teams, made up of health facility personnel within health units, were responsible for generating, reviewing, and making decisions based on HIS data. PMTs were expected to meet monthly to review routine facility data, identify areas for improvement, and take corrective action. To strengthen the PMTs, the MOH and its partners provided in-service training and frequent supervision to equip and empower health staff to effectively use data for monitoring program performance.^9^

### Health Information System Digitization

IR pillar 2 centered on deploying HIS technology and creating a supportive policy environment for scaling and sustainability. In 2015, the MOH transitioned from 2 legacy HMIS systems to a single system supported by DHIS2,^21^ an open-source, web-based HIS platform.^9^ To facilitate the DHIS2 rollout and encourage the use of its online version, the MOH expanded high-speed Internet coverage and provided information technology support to facilities. An electronic master facility registry was also introduced to capture, store, and share information on health facilities nationwide. The MOH also deployed an electronic medical records system—SmartCare EMR—in selected health facilities to harmonize data across platforms. In 2018, the CHIS was upgraded to an electronic system (eCHIS) and launched as a mobile platform with additional modules like the digital family folder.

### Health Information System Governance and Leadership

From 2017 to 2019, Ethiopia established an electronic health architecture (eHA) that addressed software, hardware, system interactions, and HIS governance needs at national and regional levels.^5^ Simultaneously, a HIS governance framework was developed, outlining governance functions, principles, and structures. Additional governance documents were created to address data access and sharing guidelines, requirements, and standards for interoperability across systems like the master facility registry, National Health Data Dictionary, and electronic medical records.

To monitor HIS progress and promote coordination and stewardship, the HIS Steering Committee, National HIS Advisory Group, and technical working groups focusing on data use, digital health, and governance were established. Exchange platforms such as the Annual Review Meeting, Joint Steering Committee, and PMTs were also formed to align partner engagement around HIS. Furthermore, the National Health Data Directory and National Classification of Diseases were deployed to promote system harmonization.

## EARLY EFFECTS OF THE INTERVENTIONS

At the end of the HSTP-I, significant progress was made in HIS digitalization and governance. By 2021, all public facilities had access to DHIS2, with 71% of these facilities using it online. Additionally, 3,605 health institutions were connected to the Health Net virtual private network, and more than 7,000 health posts had access to the eCHIS.^20^ Governance structures were operational at both the national and regional levels, including key partnership forums, the rollout of various HIS policies and guidelines, and the deployment of interoperable information communication technology systems to manage human resources, health records, commodities, and disease surveillance.^20^

A review of the literature on IR interventions reveals improved data quality and use during the early implementation phase (2018–2022). Belay et al.^8^ conducted a baseline and midline PRISM assessment in 24 “learning and demonstration” facilities that participated in the CBMP program. These sites received a more intensive package of HIS interventions during the program period, including frequent supportive supervision and mentorship visits and additional in-service trainings on data quality and use. The study found significant improvements in data quality from baseline to midline. For example, quarterly reporting completeness improved from 26% to 83%, submission timeliness rose from 17% to 48%, and data accuracy between source documents and reported data improved across key tracer indicators.^8^ Belay et al. also reported better data use in these facilities between baseline and midline, particularly through the establishment and use of PMTs and the application of data in planning and target setting, problem-solving, and action plan development.

Early evidence shows improved data quality and use and progress in HIS digitization and governance.

Other studies that assessed CBMP facilities garnered similar results. In nearly all facilities participating in the CBMP (174 health centers, 35 hospitals, and 30 woreda health offices), HIS infrastructure, data quality, and use practices increased over the course of the program.^8^^,^^17^^,^^22^^–^^24^ In a study conducted by Tilahun et al. that compared HIS performance, as measured through the Connected Woreda checklist before and after interventions, showed that HIS performance improved across all measured domains—HIS infrastructure, data quality, and administrative data use.^24^ The study also noted that many of the assessed health units improved their Connected Woreda classifications during the implementation period, with more than 30% of health units moving from emerging or candidate status to model status by endline.^24^

Several studies highlight the crucial role of a strong PMT in improving data quality and utilization. Research during the implementation of DUP consistently showed that active PMT involvement was associated with better data quality and management at the facility and woreda levels.^8^ In a study exploring the drivers and barriers of improved data quality and use in 3 subcities of Addis Ababa, the authors found that the strengthened PMTs, along with increased capacity-building and mentorship opportunities, were paramount to ensuring data quality.^10^ They also found that committed leadership and ongoing engagement drove improved use of routine data. Another study found that PMTs facilitated a structure for peer-to-peer capacity-building and performance-based recognition, enhancing data use.^25^ In the Harari region, research in 42 public health facilities showed that departments receiving regular feedback from PMT teams were more likely to have high-quality data compared to those who did not receive regular feedback (adjusted odds ratio: 3.083; 95% confidence interval=1.549, 6.135).^12^ A study in Oromia also demonstrated that the provision of feedback, regular supervision, and managerial support was associated with increased commitment to use DHIS2 data.^26^

Challenges and lessons learned were also noted in the literature and in dissemination reports from DUP. Implementors from DUP noted issues with system interoperability, frequent updates to DHIS2, and the resource demands of achieving comprehensive coverage with eCHIS.^27^ Problems with integrating eCHIS and DHIS2 resulted in fragmented reports and data inconsistencies. Additionally, the large volume of daily data and misalignment with the eHA further complicated these issues.^28^

Persistent obstacles in fostering a culture of data use were also noted. Belay et al. found that while PMTs were established in many facilities, the frequency of monthly meetings declined between baseline and midline,^8^ though this decline was partly due to the COVID-19 pandemic and social distancing practices. The study also highlighted the need for improved supportive supervision practices at the primary health care level to boost data quality and engagement. The study concluded that ensuring consistent coordination with local HIS leaders and academic partners, providing focused support to facilities, and establishing regular monitoring mechanisms for HIS improvements are crucial to improving HIS performance.

## DISCUSSION

In 2016, Ethiopia launched an ambitious agenda to strengthen its HMIS and improve the quality and use of data generated by its system. From 2016 and 2022, the MOH and partners advanced this agenda by moving to a single integrated HIS platform, establishing an eHA to foster harmonization, and strengthening HIS governance, coordination, and leadership. Efforts also focused on improving data quality and use by strengthening mechanisms, like PMTs, and increasing capacity-building with targeted training, mentorship, and supportive supervision. We aimed to highlight the early effects of the IR interventions on HIS performance as documented in the literature.

The interventions most cited as facilitators for improved data quality and use in the literature include functioning PMTs, capacity-building and mentorship activities, enhanced HIS technologies, and leadership engagement.^8^^,^^10^^,^^29^ A study conducted across the 3 DUP projects in Ethiopia, Ghana, and Mozambique derived similar facilitators (i.e., skill-building collaborations, peer-to-peer exchanges, mentoring, and leadership engagement) for HIS performance across all 3 programs.^29^ These findings are further echoed in the literature on HIS performance in low- and middle-income countries. Many of these studies and reviews have found that the combination of technology enhancement with strengthened capacity-building activities, feedback mechanisms, and interventions that facilitate data availability all lead to improved data use for decision-making.^22^^,^^30^^–^^33^

The interventions most cited as facilitators for improved data quality and use include functioning PMTs, capacity-building and mentorship activities, enhanced HIS technologies, and leadership engagement.

Despite the achievements of the HSTP-I and IR, Ethiopia faces enduring HIS challenges that must be addressed to realize its vision of a culture of data use and evidence-based decision-making. Challenges noted during the implementation of DUP included difficulties in achieving system interoperability that resulted in disrupted data reporting and use, persistent fragmented reporting, and data inconsistencies that hindered effective decision-making. The MOH has taken steps to improve system alignment by engaging stakeholders from various sectors and developing detailed use cases to improve interoperability. These efforts are critical for sustaining advancements in HIS digitalization and governance and fostering a unified system capable of supporting long-term health outcomes.

Efforts to improve system alignment and reduce fragmented reporting are underway, but these broader systemic issues trickle down to individual-level challenges. Health workers continue to struggle with accessing, analyzing, and effectively using data for decision-making. Despite improvements in data quality, certain dimensions remain inadequate, and capacity for data use is still low in many areas of the country.^15^ While promising data use interventions have been implemented throughout the country, information use for planning and decision-making is inconsistent across the health system. To apply health data consistently and effectively for planning and managing health programs, data must be accessible and able to be processed into meaningful formats through analysis and visualization. Barriers, such as inconsistent Internet access and limited data analysis, interpretation, and problem-solving skills among health workers, continue to hinder progress in Ethiopia.^10^

The shortage of a skilled HIS workforce has also been a persistent challenge.^10^^,^^29^ A recent study by DUP and Gondar University found that only 5% of health institutions have an adequate number of HIS staff in place according to MOH standards.^34^ This study also highlighted the need to expand the HIS workforce to 50,000 workers by 2030 to meet national needs. Similar issues have been observed in other countries, including shortages of trained personnel, limited data accessibility, and low health worker motivation.^31^^,^^35^^,^^36^ These challenges can have far-reaching implications on the delivery of quality health care services, which is why documenting the progress of HIS interventions and their effect on HIS performance is critical in moving Ethiopia toward its vision of sustained health system performance through the effective use of quality data for decision-making.

## CONCLUSION

Under the IR, the MOH and its partners have made significant strides in improving HIS infrastructure and fostering a culture of data use, with studies showing promising improvements in data quality and data use across health facilities in Ethiopia. To ensure sustainable progress, it will be crucial to continue addressing key challenges, including strengthening system interoperability, expanding the HIS workforce, and enhancing capacity for data use at all levels. By building on the successes of the first HSTP-I and addressing these gaps, Ethiopia can further its vision of a robust, data-driven health system capable of improving health outcomes and driving evidence-based decision-making.

## Notes

**Peer Reviewed**

**First Published Online:** November 4, 2024.
